# An improved plastination method for strengthening bamboo culms, without compromising biodegradability

**DOI:** 10.1038/s41598-023-32662-w

**Published:** 2023-04-06

**Authors:** Reeghan Osmond, Olivia H. Margoto, Ibrahim Alper Basar, Tina Olfatbakhsh, Cigdem Eskicioglu, Kevin Golovin, Abbas S. Milani

**Affiliations:** School of Engineering, University of British Columbia, Kelowna, BC V1V1V7 Canada

**Keywords:** Engineering, Materials science

## Abstract

Biomaterials are increasingly being designed and adapted to a wide range of structural applications, owing to their superior mechanical property-to-weight ratios, low cost, biodegradability, and CO_2_ capture. Bamboo, specifically, has an interesting anatomy with long tube-like vessels present in its microstructure, which can be exploited to improve its mechanical properties for structural applications. By filling these vessels with a resin, e.g. an applied external loading would be better distributed in the structure. One recent method of impregnating the bamboo is plastination, which was originally developed for preserving human remains. However, the original plastination process was found to be slow for bamboo impregnation application, while being also rather complicated/methodical for industrial adaptation. Accordingly, in this study, an improved plastination method was developed that is 40% faster and simpler than the original method. It also resulted in a 400% increase in open-vessel impregnation, as revealed by Micro-X-ray Computed Tomography imaging. The improved method involves three steps: acetone dehydration at room temperature, forced polymer impregnation with a single pressure drop to − 23 inHg, and polymer curing at 130 °C for 20 min. Bamboo plastinated using the new method was 60% stronger flexurally, while maintaining the same modulus of elasticity, as compared to the virgin bamboo. Most critically, it also maintained its biodegradability from cellulolytic enzymes after plastination, as measured by a respirometric technique. Fourier transform infrared-attenuated total reflection, and thermogravimetric analyses were conducted and showed that the plastinated bamboo’s functional groups were not altered significantly during the process, possibly explaining the biodegradability. Finally, using cone calorimetry, plastinated bamboo showed a faster ignition time, due to the addition of silicone, but a lower carbon monoxide yield. These results are deemed as a promising step forward for further improvement and application of this highly abundant natural fiber in engineering structures.

## Introduction

Biomaterials have been quickly gaining popularity for use in structural applications due to their low cost, high strength-to-weight ratio, processibility, biodegradability, and low toxicity^1^. This has inspired a search for strengthening methods that allow biomaterials to be used in broader structural applications, replacing non-renewable and petroleum-based materials.

Bamboo is one of the world’s fastest growing plants, maturing in only 3–4 years and is native to all continents other than Antarctica and Europe^2,3^. Because of this, bamboo plays an important part in atmospheric CO_2_ balance through CO_2_ sequestration and O_2_ production^2^. Additionally, since bamboo can grow in poor soil, it can be used for ecological and soil restoration^2^. Thus, by using bamboo and other sustainable biomaterials in more structural applications, they will be planted and grown more often to sequester CO_2_, produce O_2_, and restore soil.

According to Liese et al.^4^, the bamboo culm is comprised of around 50% parenchyma, 40% fibres, and 10% vascular bundles. The most elongated vessels are within the vascular bundles, where there are two metaxylem vessels and metaphloem of sieve tubes. Specifically, the metaxylem vessels are long cells that are connected by the large perforations allowing material to flow within them more easily. Hypothetically, some of these elongated and perforated cells can be filled with a polymer resin to create a composite with improved mechanical properties by distributing the applied load^5^.

Plastination, originally invented to preserve human remains for teaching anatomy^1,6^, has recently been deemed as a novel method for impregnating some of the vascular tissues in bamboo with a polymer, like silicone resin^7^. The original plastination method increased the bamboo’s tensile strength and modulus by 70% and 20%, respectively, while only partially impregnating the cells^7^. However, the standard S10 plastination method is known to be slow and methodical process that focuses on carefully preserving the anatomical structures and size of the human tissue specimens. Bamboo, on the other hand, is much stronger and preservation of its exact anatomy is not critical when its end usage is in engineering/industrial applications.

Thus, the aim of the present work is to improve the standard S10 plastination method by redesigning each step of the process to be specifically adapted to bamboo. Namely, improvements in speed of the plastination process, the simplicity of each step, and the amount of silicone resin impregnated within the bamboo were pursued. After plastination with the improved method, flexural, thermal, flammability and biodegradation tests were performed, as they are common performance measures considered for bamboo in structural applications.

## Materials and methods

### Materials

Yellow stripe timber bamboo (*Phyllostachys Bambusoides*) was provided by Canada’s Bamboo World located in Chilliwack, BC, Canada (in compliance with all ethical measures). Two types of silicone resins were used: a three-part silicone resin that cures at room temperature and a one-part silicone resin that cures with heat. The three-part silicone resin, used in the standard method^6^, consisted of NCS10, NCS6, and NCS3 and was obtained from Silicones, Inc. in High Point, NC, USA. The NCS10 and NCS6 were mixed (10:1 ratio by volume^6^) and used in the forced polymer impregnation step. NCS3 was used as a spray-on curing agent. Used in the improved method, the one-part silicone resin, called SS-151, was acquired from Silicone Solutions (Cuyahoga Falls, OH, USA). SS-151 is a self-leveling RTV that cures at 130 °C in 20 min and has a working time of 7 or more days. The 99.5% acetone used in the process was acquired from Avantor in Radnor, PA, USA.

### Standard S10 plastination method

The standard S10 plastination method for treating bamboo has three steps: acetone dehydration, forced polymer impregnation, and polymer curing^7^. The forced polymer impregnation set-up here involved a TRIVAC AR4-8 vacuum pump that was connected directly to the vacuum chamber. The 5-gal vacuum chamber and connections were purchased from McMaster-Carr (Robbinsville, NJ, USA).

Bamboo specimens were first cut into the desired geometry using a waterjet cutter. To ensure that the waterjet only cut through one side of the bamboo at a time, a metal rod was inserted into the culm for protection. Then, the specimens were placed in 99.5% acetone at − 25 °C for three days to complete the acetone dehydration step. The specimens were stirred daily.

Next, the specimens were removed from the acetone and quickly placed in a container holding the mixture of NCS10 and NCS6 silicone resins, all within a vacuum chamber. The pressure within the vacuum chamber was incrementally dropped to − 29.5 inHg. Between each increment, the samples were monitored, through the glass top of the vacuum chamber, for bubble formation from the longitudinal openings of the bamboo. Once the bubbles, composed of acetone vapour and other substances like trapped air, stopped forming, the pressure was decreased to the next increment. The first increment was from atmospheric pressure to − 17.5 inHg. Afterwards, the pressure was decreased by 4 inHg until − 29.5 inHg was achieved. The pressure was then released, and the samples were left in the chamber for six hours.

Finally, the specimens were removed from the silicone resin mixture and blotted with a paper towel. The specimens were sprayed with NCS3 using a spray bottle and covered with plastic wrap. The excess mixture was blotted with a paper towel again before another layer of NCS3 was sprayed onto the specimens 24 h later. They were then wrapped with plastic wrap again and left to cure for another 24 h. Afterwards, the plastic wrap was removed from the specimens for evaluation.

### Improved plastination process: trials

#### Initial trials

Different experiments were conducted to improve each step of the standard plastination process outlined above, specifically for bamboo. First, improvements to acetone dehydration were investigated by performing this step at different temperatures. Then, improvements to forced polymer impregnation were investigated by performing this step at different pressures, speeds, and temperatures. Between the two sets of experiments, the silicone resin used was changed from the standard three-part silicone to the one-part SS-151 (which is a heat cure silicon). A summary of the improvement experimental trials performed is provided in Table 1.

**Table 1:** Summary of improvement trial experiments performed and comparison to the original S10 plastination method.

Step in focus of improvement/optimization	Purpose of experimental trials	Experiment #	Steps followed in improvement experimental trials			Steps in original S10 method
			Acetone dehydration	Forced polymer impregnation	Polymer curing	
Acetone dehydration	Simplify step by removing requirement for freezer	1	− 25 °C for 3 days	Incremental decrease in pressure to − 29.5 inHg at 20 °C	NCS3 spray-on curing	− 25 °C for 3 days
		2	5 °C for 3 days			
		3	20 °C for 3 days			
Polymer curing (polymer selection)	Simplify and speed-up step by changing from a three-part spray-on curing to one-part heat curing polymer	4	20 °C for 3 days	Incremental decrease in pressure to − 29.5 inHg at 20 °C	SS-151 post-oven curing at 130 °C for 20 min	NCS3 spray-on curing
Forced polymer impregnation	Simplify and speed-up step by either removing the need for a vacuum chamber or removing the need for incremental decreases in pressure within chamber	5	20 °C for 3 days	Atmospheric pressure (no vacuum chamber) at 60 °C	SS-151 cured during forced polymer impregnation step	Incremental decrease in pressure to − 29.5 inHg at 20 °C
		6		Immediate decrease in pressure to − 29.5 inHg at 20 °C	SS-151 post-oven curing at 130 °C for 20 min	
		7		Immediate decrease in pressure to − 23 inHg at 20 °C		

Note that, although polymer curing is the last step of the entire process, it needed to be optimized (i.e. selecting an alternative resin and recommend its curing profile) prior to the forced polymer impregnation trials, as the latter must use the same selected polymer.

When investigating the effects of temperature on acetone dehydration, the bamboo specimens were cut into the 4 mm × 110 mm × 0.1 to 0.5 cm (transversely × longitudinally × radially) sized rectangular pieces and weighed. The thickness of the specimens varied from 1 to 5 mm depending on the thickness of the original culm. Three alternative recipes were performed as follows.

- The first set of samples was submerged in a 0.5 L bottle of acetone and placed in a freezer at − 25 °C for three days, following the standard plastination method.
- The second set of samples was placed in a different 0.5 L bottle of acetone and were held at 5 ºC for three days in an environmental chamber (S/SM Series from Thermotron in Holland, MI, USA).
- The final set of samples was placed in a 0.5 L bottle of acetone at room temperature (20 ± 2 °C) for three days. All of the samples were then plastinated following the remaining steps in the standard S10 plastination method. The samples were then weighed and cut in half to be scanned using EDS to find their Si content. The lowest Si content was expected to be at the centre of the bamboo ^7^.

Regarding the polymer to be used in steps 2 and 3 of the process, it was changed from a multi-part, spray-on resin that cures after several days (i.e. NCS3 in the standard plastination) to a one-part, heat curing resin (silicone resin SS-151) that can cure after as lows as 20 min (at 130 °C). This selection/improvement for the polymer curing step was decided before the forced polymer impregnation improvement trials (Table 1); as the characteristics of the selected polymer directly affects the forced impregnation parameters.

For the forced polymer impregnation improvements, the bamboo samples were cut into 1 cm × 3 cm × 0.1–0.5 cm rectangular sized pieces to conserve material. After acetone dehydration, the bamboo samples were quickly removed and placed in a container of the uncured SS-151 resin. The samples were held down with a binder clip to stop them from floating. The vacuum was then applied, and vacuum impregnation was deemed complete when bubble formation, and release from within the bamboo, ceased. Three alternative recipes were performed as follows.

- The first forced polymer impregnation improvement trail was conducted to investigate the effect of temperature. Acetone boils at either 56 °C at atmospheric pressure ^8^ or at approximately – 20.5 inHg at 25 °C ^9^. Thus, the container of bamboo and SS-151 resin were placed in the Thermotron environmental chamber at 60 °C.
- The second forced polymer impregnation improvement trail was conducted to investigate the effect of quickly decreasing the pressure. In this experiment, the container of bamboo and SS-151 resin was placed in the vacuum chamber and the pressure was dropped to − 29.5 inHg directly, instead of incrementally as in the standard S10 plastination method ^6^.
- The last experiment was also used to investigate the effect of pressure drop speed. However, this time the pressure was quickly decreased to − 23 inHg instead of dropping fully to – 29.5 inHg, since acetone boils at around − 20.5 inHg at 25 °C ^9^.

#### Final selected (optimal) plastination process

**Figure 1:**
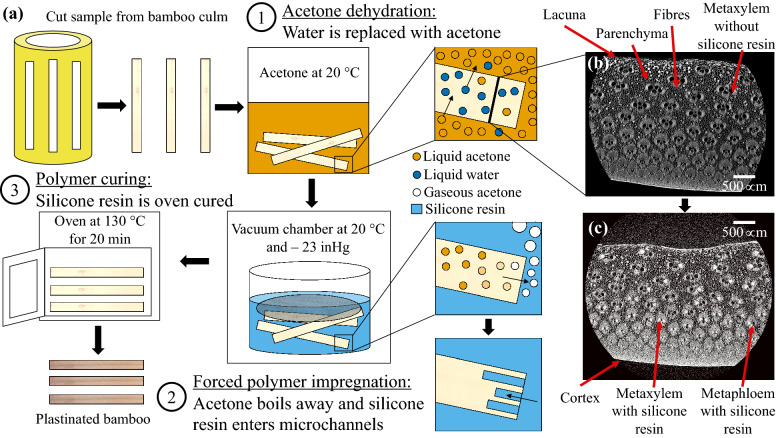
(**a**) The three main steps in the improved (final recommended) plastination method: acetone dehydration, forced polymer impregnation, and polymer curing. Micro-CT images on the right show the microstructure of a bamboo specimen’s cross-section (**b**) before and (**c**) after plastination.

The final improved plastination steps (Fig. 1a) were devised based on the results of the individual experimental trials outlined in section "Initial trials". The process still involves three main steps: acetone dehydration, forced polymer impregnation, and polymer curing.

Step 1: During acetone dehydration, bamboo was submerged in 99% acetone for three days at room temperature (20 ± 2 °C). Approximately 0.5 L of acetone was used per 3 g of bamboo. The acetone and bamboo were either placed in a large sealable polypropylene container or in a small sealable glass bottle. Almost all of the bamboo pieces were denser than the acetone and sunk to the bottom of the container immediately. There were a few pieces that did not sink and were weighed down by glass or a piece of polypropylene to ensure that they did not dry at the surface of the liquid. Each day, the samples were stirred in the acetone.

Step 2: Directly after acetone dehydration, the bamboo was removed from the acetone and placed in a container with the SS-151 silicone resin. The bamboo samples were held down by either a mesh or binder clips to ensure that they did not float and were covered with the silicone resin. The container was then placed in the vacuum chamber set-up described in section "Standard S10 plastination method", which allowed for the samples to be visually monitored. A – 23 inHg pressure was applied immediately without increments, using the vacuum pump. The samples were periodically observed for bubble formation. After all the bubbles subsided (after approximately 42 h), the pressure was increased back to atmospheric. The samples were then left in the silicone resin for six hours to ensure that the polymer filled the bamboo’s vessels.

Step 3: Finally, the samples were removed from the silicone and wiped with paper towels. They were then placed in an oven to cure for 20 min at 130 °C. The silicone’s viscosity decreases as the temperature is increased, and so to avoid leakage, the longitudinal ends were blocked-off while in the oven. To do this, the bamboo samples were placed between two metal blocks that were clamped into place. Double-sided vacuum bagging tape was placed between the metal blocks and the ends of the samples.

As seen in Fig. 1b, the cross-section of bamboo consists of many hollow tube-like structures called metaxylem that transport water^4^. After plastination, these metaxylem were filled with the silicone resin (Fig. 1c).

### Micro X-ray computed tomography

A Zeiss/Xradia X-400 Micro- X-ray computed tomography (Micro-CT) device (Dublin, CA, USA) was used to verify the amount of silicone resin present in the bamboo specimens upon plastination. It was also used to investigate the microstructure of the specimens and look for cracks that formed during the plastination process. All of the scans used a voltage of 40 kV, a current of 500 mA, and the 4 × objective. The scans all had a pixel size in the range of 2.99–3.17 μm. There were 2500 projections taken over 360° and Avizo (version 9.3.0) by Thermo Fisher Scientific in Waltham, MA, USA was used to analyze the scans and generate images.

### Energy dispersive X-ray spectroscopy

A Tescan (Brno, Czech Republic) Mira 3 XMU scanning electron microscope (SEM) was used to image and perform energy dispersive X-ray spectroscopy (EDS) on the centre of plastinated bamboo specimens. With this, the Si content at the centre of different specimens was measured. A 20 nm layer of platinum was first sputter coated onto the bamboo to reduce charging effects.

### Flexural tests

Using the Instron 5969 (Norwood, MA, USA) dual column universal testing system with a 50 kN load cell, three-point bending tests (following ASTM D790^10^) were performed on six plastinated and six non-plastinated bamboo specimens to measure the flexural strength and tangent modulus of elasticity (MOE). The specimens had nominal dimensions of 4 mm × 110 mm and were cut from culms using a waterjet cutter. The thickness of the specimens varied anywhere from 1 to 5 mm. Because of this large thickness variation, the speed and support span were changed between each test. The support span was adjusted such that it was always 16 times the thickness of each sample.

### Biodegradability

The biodegradability index of plastinated and non-plastinated bamboo was calculated by dividing the average specific oxygen uptake (SOU)^11^ value from the average chemical oxygen demand (COD)^12^ value.

A PF-8000 Pulse Flow Respirometer (from Respirometer Systems and Applications in Fayetteville, AR, USA) was used to perform the SOU test (conducted at 36 °C). In total, eight channels were used to measure the oxygen demand of different samples in this test. There were two non-plastinated bamboo channels, two plastinated bamboo channels, two positive controls, and two inoculum channels (blanks). All the reactors contained macro–micro nutrients and a buffer (KH_2_PO_4_) to maintain a pH of 7. The plastinated and non-plastinated bamboo channels contained cellulolytic enzyme-rich inoculum and the bamboo samples.

The base of the inoculum was Ogogrow compost and was obtained from the Regional Composting Facility in Vernon, BC, Canada. The Ogogrow compost, rich in white rot fungi, was made from biosolids and hog fuel, and its C:N ratio was 10.8. Due to the hog fuel used in the compost, the microbial consortium of the compost was suitable to break down the lignocellulosic structure. The compost was kept at 55 °C for three months in an aqueous environment to enrich the inoculum media in cellulolytic enzymes. Following this, the inoculum was aerated for 10 days in a beaker, where it was fed with microcrystalline cellulose to promote enzyme production before being used in the SOU test.

At the end of the SOU test, the oxygen consumption of each sample was calculated by subtracting the average amount of oxygen consumed by the blanks. These values were then normalized to 0 °C and atmospheric pressure. This test was deemed complete after 10 days, which was when the reactors with bamboo samples appeared to stop consuming oxygen.

5 g of plastinated and 5 g of non-plastinated bamboo were cut into approximately 5 mm length pieces from their original size (4 mm × 110 mm). 2.5 g pieces of plastinated bamboo were placed in each of the two plastinated bamboo channels and 2.5 g pieces of non-plastinated bamboo were placed in each of the non-plastinated bamboo channels.

To measure the COD, three plastinated and three non-plastinated bamboo samples were first broken into 1.2 mg pieces before being digested with H_2_SO_4_ under the catalytic effects of Ag_2_SO_4_ and HgSO_4_. Digestion was performed in a temperature chamber (S-3200 from Thermotron in Holland, MI, USA) at 150 °C for 3 h. The amount of oxygen consumed per gram of sample during the digestion was measured using a spectrophotometer (GENESYS 10S Series from Thermo Fisher Scientific in Waltham, MA, USA) at a 600 nm wavelength.

The total solids (TS) and volatile solids (VS) contents were found following the EPA EPA-821-R-01–015 method 1684^13^. Two pieces of non-plastinated bamboo and two pieces of plastinated bamboo (1 cm × 3 cm × 0.1–0.5 cm) were first weighed and then placed in an oven for 24 h at 105 °C. After drying, they were weighed again to get the TS and moisture content of the samples. After this, the samples were burned at 550 °C for 2 h and weighed again to get the ash content and VS of the samples.

### Fourier-transform infrared spectroscopy

Fourier transform infrared-attenuated total reflection (FTIR-ATR) was used to characterize the functional groups of the samples using a Thermo Scientific Nicolet iS20 device from Thermo Fisher Scientific in Waltham, MA, USA. The FTIR-ATR spectra obtained were in the range of 4000 to 500 cm^−1^, at 64 scans per spectrum with a resolution of 4 cm^-1^.

### Thermogravimetric analysis

Thermogravimetric Analysis (TGA) was performed using a TGA model Q500 from TA Instruments (New Castle, DE, USA) to study the thermal decomposition behavior of cured SS-151, plastinated bamboo, and non-plastinated bamboo. Samples of approximately 13 mg were placed in a platinum crucible and heated from room temperature to 900 °C at 10 °C/min under a nitrogen atmosphere (40 mL/min).

### Flammability

Flammability tests were completed in accordance with ISO 5660-1^14^. The bamboo samples were cut into semi-cylindrical pieces with dimensions of 100 mm × 30–40 mm. When they were tested using the Cone Calorimeter, three samples were placed beside one another to meet the 100 mm × 100 mm dimensional requirements. Three 100 mm × 100 mm plastinated and three 100 mm × 100 mm non-plastinated samples were tested at a heat flux of 50 kW/m^2^. The time to ignition (t_ignition_), peak heat release rate (peak HRR, kW/m^2^), heat release rate (HRR, kW/m^2^), effective heat of combustion (EHC, MJ/kg), mass (g) loss rate, smoke specific extinction area (SEA, m^2^/kg), total smoke production (TSP, m^2^), yield of carbon monoxide (Y_CO_, kg/kg), and yield of carbon dioxide (Y_CO2_, kg/kg) were all measured. Although ISO 5660 was followed, it required that the HRR at 180 s for all specimens be within 90 to 110% of the average HRR at 180 s. Not all of the plastinated samples had HRR that fell within this range.

## Results and discussion

### Improvements to the standard S10 plastination method

**Figure 2:**
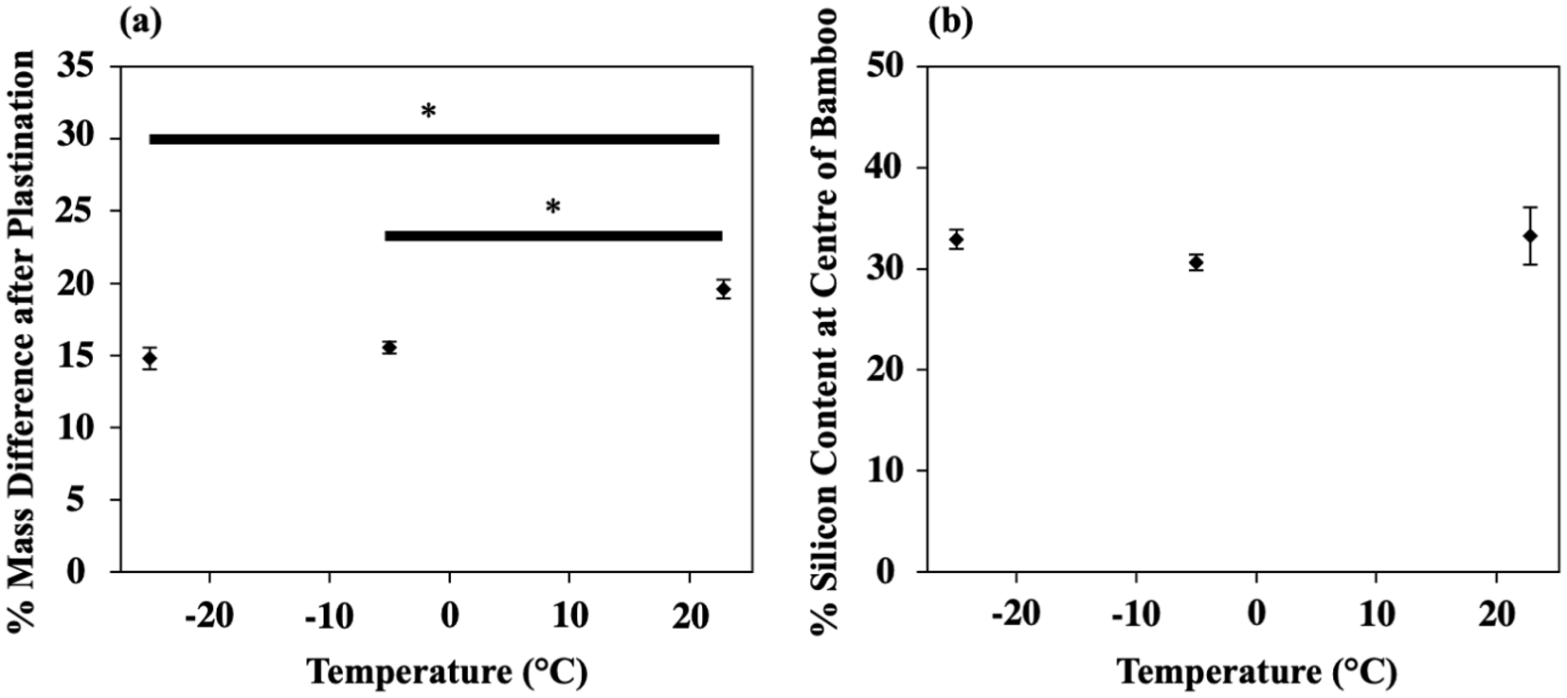
The increase in (**a**) mass and (**b**) silicon content (measured with EDS) at the centre of bamboo specimens after plastination, that were dehydrated at different temperatures. *Significance level of < 0.05 (One-way ANOVA).

As expected, the bamboo samples all increased (up to 30%) in mass after plastination using the different acetone dehydration temperatures (Fig. 2a). The specimens that underwent acetone dehydration at room temperature gained the most silicone mass overall. The viscosity of liquids decreases with temperature, meaning that the speed of acetone flow into the bamboo’s vessel and water substitution would likely increase at higher temperatures. Figure 2b shows that the silicon content at the centre of all the bamboo specimens was the same regardless of the acetone dehydration temperature. Completing acetone dehydration at different temperatures, therefore, did not improve the depth of silicone resin impregnation. This was likely because the required dehydration time used (three days) was the minimum time required for − 25 °C; thus, it was sufficient for the higher temperatures as well. The forced polymer impregnation step required improvement to change the impregnation depth. However, since acetone dehydration at room temperature resulted in the most silicone mass gain and did not require a freezer or environmental chamber to maintain a lower temperature, it was deemed advantageous compared to the original − 25 °C condition.

Since bamboo is stable at temperatures less than 200 °C ^15,16^, unlike human remains, a quick, heat-curing cycle like that required for the SS-151 was deemed more reasonable. Using SS-151 instead of NCS10, NCS6, and NCS3 was also simpler because it did not require a by-volume mixture or multiple, long spray-on curing cycles. Although SS-151 was selected because it cures at 130 °C in 20 min and is stable at room temperature, this did not mean that it would not slowly cure at elevated temperature. When forced polymer impregnation was completed at 60 °C without vacuum, above the boiling point of acetone, the SS-151 silicone cured before impregnation was completed. There were bubbles cured into the SS-151 that were trapped as they flowed from the bamboo specimen. Thus, the vacuum chamber was still required to complete forced polymer impregnation using the SS-151 silicone resin.

**Figure 3:**
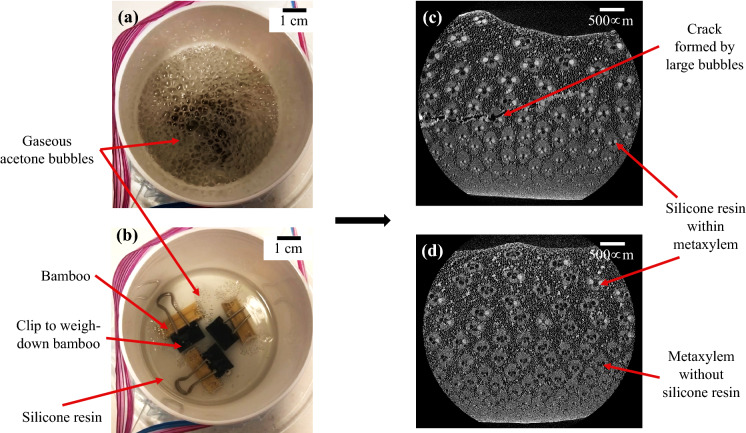
Acetone boiling away from within the bamboo specimens during forced polymer impregnation at (**a**) high vacuum pressure and (**b**) low vacuum pressure. Micro-CT images showing (**c**) a crack in a bamboo specimen due to large bubbles and (**d**) only some of the metaxylem filled with silicone after incomplete forced polymer impregnation.

During forced impregnation, decreasing the pressure from atmospheric to − 29.5 inHg without increments was investigated because the specimens did not require continual observation between increments to witness when the bubble formation stopped. By decreasing the pressure immediately, the end of bubble formation only had to be observed once. However, after the pressure was decreased to − 29.5 inHg without increments, large aggressive bubbles formed (Fig. 3a). Using Micro-CT, cracks were observed at the centre of the bamboo specimens from this experiment (Fig. 3c). Normally, the bubbles seen at lower vacuum pressures appeared like those seen in Fig. 3b and did not cause cracking but may result in incomplete impregnation (Fig. 3d). Therefore, this pressure ramp was deemed too high.

By decreasing the pressure only to − 23 inHg, without increments, no cracks were formed in the bamboo samples. The pressure of − 23 inHg was chosen because it was sufficiently low to remain under the boiling point of acetone at room temperature (− 20.5 inHg), yet not low enough to cause any internal cracks. As the acetone boiled from inside the samples, the overall pressure in the chamber did increase. However, for the amount of bamboo and chamber size used in this work, the pressure still remained below the boiling point of acetone at room temperature during the process. It is possible that if a much larger number of specimens were plastinated, and thus a larger volume of acetone was boiled off, the pressure could rise above − 20.5 inHg, which would stop the impregnation process prematurely.

To ensure that the silicone resin had time to reach the centre of the bamboo specimens, they were left in the vacuum chamber for an additional six hours. If the bubbles were not allowed to fully subside before releasing the vacuum and the specimens were removed prematurely (after a few minutes), only a few metaxylem vessels at the centre of the bamboo would fill. This resulted in incomplete impregnation, similar to what was observed when reaching a low vacuum pressure (Fig. 3d).

**Figure 4:**
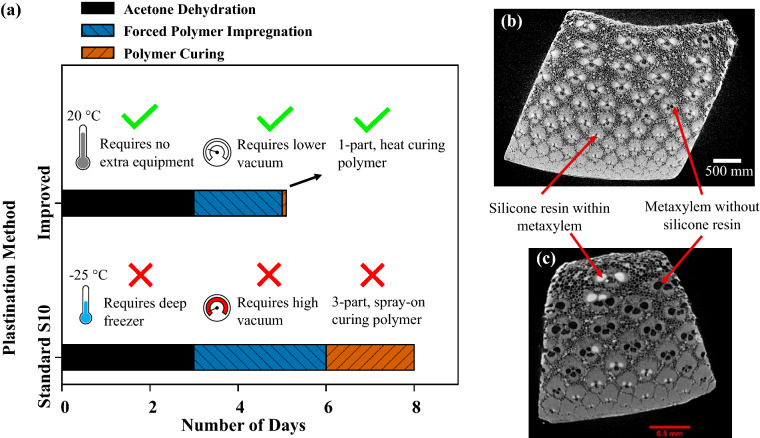
(**a**) Comparison between the processing time taken to plastinate bamboo using the standard S10 and the improved methods. A Micro-CT 2D image slice comparison between the number of metaxylem vessels filled at the centre of a bamboo specimen plastinated using the (**b**) improved and (**c**) standard S10 method ^7^.

From Fig. 4a, overall, the improved plastination method was simpler and 40% quicker than the standard S10 method, taking only 5 days. The improved process did not require the specimens to be cooled during acetone dehydration, did not require a high vacuum or continuous monitoring, and used a one-part silicone resin that cures quickly with heat.

Using the software ImageJ (version 1.8.0_172) from the National Institute of Health (Stapleton, New York, USA) to analyze Micro-CT images, it was found that 90% of the area within the metaxylem were filled with silicone resin using the improved method (Fig. 4b) as opposed to the only 20% (Fig. 4c) achieved in a previous study using the standard S10 method^7^. By visually dissecting the 3D Micro-CT images, namely to generate the 2D slices in Fig. 4b and 4c, it was observed that no parenchyma and not many sieve tubes were filled with silicone resin using either method. This indicated that only the vessels open at either end of the bamboo sample, or those with large perforations, could be impregnated with silicone resin using plastination.

Some traditional methods for treating bamboo, such as steeping, soaking, and vertical soak and diffusion can take upwards of 7–14 days to complete^4^. Other treatment methods, such as polyethylene glycol impregnation and paraffin heat treatment that protect bamboo from cracking, can take almost three weeks^17^. This includes a two-week acetone soaking step and various soaking, heating, and drying steps^17^. To obtain transparent bamboo, impregnated with a three-part epoxy, five steps are required, including a microwave step, vacuum impregnation, and creating a solution with a precise pH^18^. Thus, the improved plastination method with its five-day three-step process may be on par with or simpler/faster than other treatment methods.

### Flexural properties

**Figure 5:**
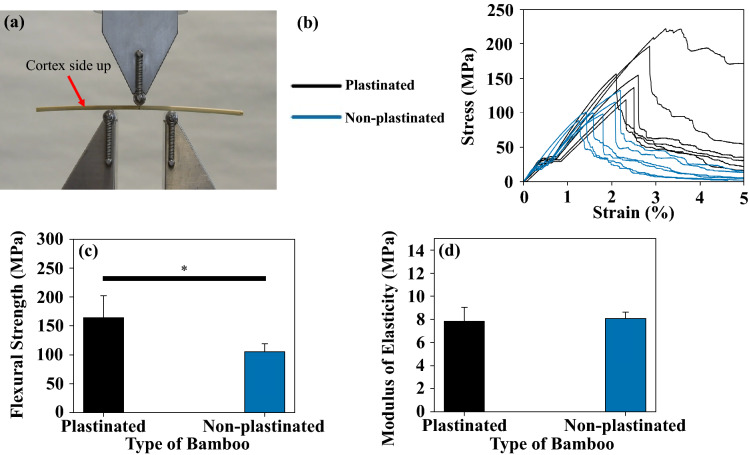
(**a**) Bamboo sample in three-point bending set-up with the cortex side facing upwards. (**b**) Stress vs. strain measurements of the plastinated and non-plastinated bamboo specimens. (**c**) Flexural strength and (**d**) tangent modulus of elasticity of the bamboo specimens. *Significance level, *P* value < 0.05 (One-way ANOVA).

The mounting orientation of the bamboo specimens and the stress versus strain data from the flexural tests can be seen in Fig. 5a,b. A summary of the average flexural strength and average tangent MOE of the samples can be found in Fig. 5c,d. Dhir et al.^7^ noted a 70% increase in the tensile strength property after plastinating bamboo using the original S10 method. Although the bamboo was tested flexurally in this study, a similar trend was noted with a 60% increase in flexural strength after plastinating with the improved method (Table 2). This increase would be because the silicone in the metaxylem vessels of the samples acts as a matrix to help distribute the load ^5^. Both plastinated (with the improved method) and non-plastinated bamboo had a similar density, resulting in plastinated bamboo having a 90% higher specific flexural strength. Conversely, the specific tensile strength of S10 plastinated bamboo was 50% higher than non-plastinated bamboo^7^.

**Table 2:** Flexural strength and tangent modulus of elasticity results.

Test/sample type	Plastinated	Non-plastinated
Flexural strength (MPa)	160 ± 40*	100 ± 10*
Specific flexural strength (MPa*m^3^/kg)	0.27 ± 0.05*	0.14 ± 0.02*
Tangent modulus of elasticity (GPa)	8 ± 1	8 ± 1
Specific tangent modulus of elasticity (GPa*m^3^/kg)	0.012 ± 0.002	0.010 ± 0.001

*Significance level, *P*-value < 0.05 (One-way ANOVA).

Plastination with the improved method did not significantly affect the tensile modulus (α = 0.05). However, in Dhir et al.’s^7^ study, bamboo plastinated using the S10 method had a 20% increase in tensile modulus. Since the original S10 plastination method creates a hardened outer coating, while the improved method does not, the former specimens were stiffer. However, note that the amount of silicone impregnation also depends on the number of metaxylem vessels present in each sample. This may have been one reason why there was a larger variation in the results from the specimens plastinated using the improved method.

### Degradation behaviour of plastinated bamboo

#### Biodegradability results

**Figure 6:**
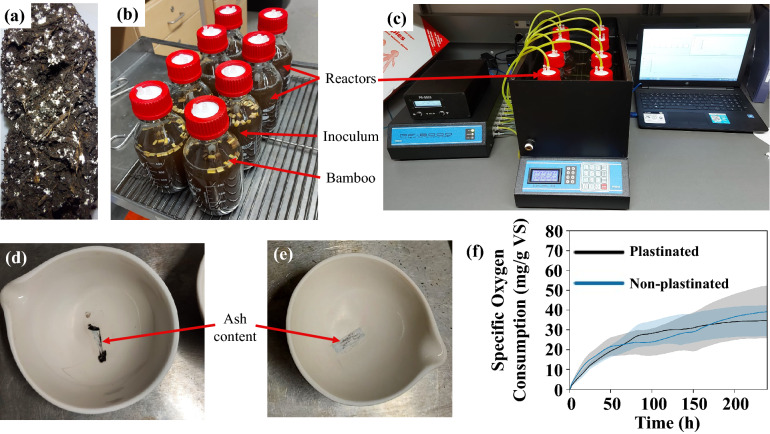
(**a**) Ogogrow compost with white rot fungi. (**b**, **c**) Reactors used in the SOU test and set-up. Residual ash content of the (**d**) plastinated and (**e**) non-plastinated bamboo present after heating for 2 h at 550 °C. (**f**) The specific oxygen consumption of the plastinated and non-plastinated samples used to calculate the aerobic biodegradability.

An image of the compost used in the experiments can be seen in Fig. 6a. In Fig. 6b,c the reactors and set-up used in the SOU tests can be found. The results from the biodegradability testing are presented in Table 3.

**Table 3:** Biodegradability test results.

Test/sample type	Plastinated	Non-plastinated
TS (g/kg)	962.9 ± 0.5*	944 ± 0.7*
VS (g/kg)	928 ± 3*	918.5 ± 0.6*
Organic matter ratio (VS/TS) (%)	96.4	97.3
Ash content (%)	3.4 ± 0.1	2.5 ± 0.4
COD (mg O_2_/g VS)	1380 ± 90	1440 ± 40
SOU (mg O_2_/g VS)	30 ± 20	40 ± 30
Biodegradation ratio (%)	2.5	2.7
Biodegradation difference (%)	8	

*Significance level, *P*-value < 0.05 (One-way ANOVA).

As expected, plastination increased the amount of solids present due to the added silicone resin (comparison in Fig. 6d,e). The average specific oxygen consumption curve, seen in Fig. 6f, was divided by VS to generate the specific values. Although the non-plastinated bamboo was 8% more biodegradable than plastinated bamboo, this was not statistically significant (α = 0.05; Fig. 6f). Results of this test indicate that cellulolytic enzymes are equally efficient on both plastinated and non-plastinated bamboo samples. Since the silicone resin was mainly present within the metaxylem vessels of the bamboo, the microbes were able to easily access and biodegrade the bamboo. The VS/TS ratio also followed this trend and revealed that there was no significant change in the organic matter ratio after plastination.

Since the main purpose of using natural fibres in structural applications is to reduce environmental impact, plastination’s ability to greatly increase the flexural strength of bamboo while maintaining its biodegradability could be extremely important when considering design for end-of-life.

#### FTIR results

**Figure 7:**
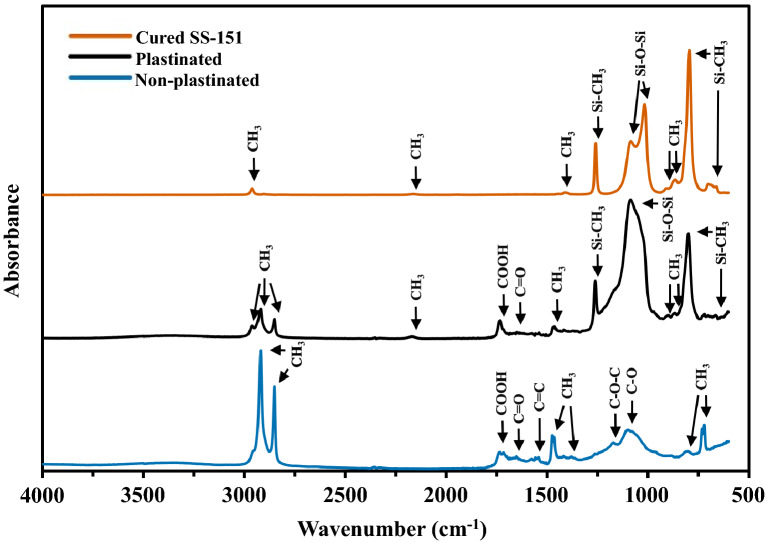
The FTIR analysis results of a cured piece of SS-151 silicone resin, a plastinated bamboo specimen, and a non-plastinated bamboo specimen.

In Fig. 7, the presence of bonds typically associated with silicones, such as Si–O–Si and Si-CH_3_, were identified in the plastinated bamboo sample^19^. The FTIR-ATR spectra showed characteristic bands attributed to the hemicellulose and lignin in bamboo, in the range of 1500–1750 cm^-1^, for both plastinated and non-plastinated samples^20,21^. The intensity of the bands attributed to C=C, C–O–C, and C–O stretching vibrations was lower for the plastinated bamboo sample. Because IR interrogates depths of only a few microns, and the bamboo was lightly covered and filled with the silicone, this decrease was not indicative of any degradation of the bamboo. Rather, the IR spectra indicated that the plastination process did not change the chemical structure of the bamboo, and only added silicone to it^20,21^. As discussed in the previous section, the biodegradation processes of the microbes were not hindered by the improved plastination method. And from the FTIR results, this may have been because the bamboo was not chemically altered by plastination.

#### TGA results

**Figure 8:**
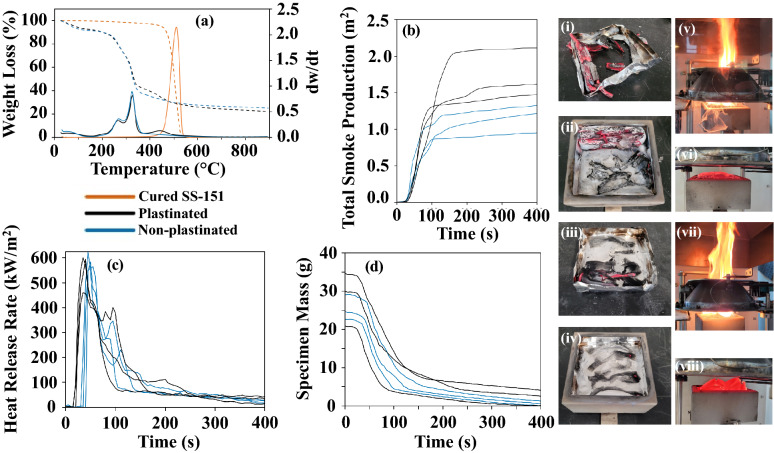
(**a**) TGA results of cured pure SS-151, plastinated bamboo, and non-plastinated bamboo. (**b**) TSP, (**c**) HRR, and (**d**) specimen mass from the cone calorimetry test (each repeated three times). (i-ii) Two of the plastinated bamboo specimens after the cone calorimetry test. (iii-iv) Two of the non-plastinated bamboo specimens after the cone calorimetry test. Large flame present after ignition for one of the (v) plastinated and (vii) non-plastinated specimens. (vi) Plastinated and (viii) non-plastinated bamboo specimens gradually burning until the end of the test.

The TGA and derivative thermogravimetry (DTG) curves of plastinated bamboo, non-plastinated bamboo, and cured SS-151 are shown in Fig. 8a and the key curve data are summarized in Table 4. The initial mass loss at 30–100 ºC may be attributed to water removal of the plastinated and non-plastinated bamboo samples. The second mass loss at 200–300 ºC mainly resulted from the decomposition of hemicellulose, and the third mass loss around 325 ºC may be attributed to cellulose and lignin decomposition, from both bamboo samples^15,16,22^. The fourth mass loss at 350–550 ºC was indicative of the presence of SS-151 in the plastinated sample, which was completed degraded at 600 ºC as shown by the DTG curve of cured SS-151. Both plastinated and non-plastinated bamboo samples had at least 20% residue remaining even at 900 °C, which can be attributed to hemicellulose and lignin^22^–^24^. Again, from the TGA results, it did not appear that plastination resulted in any significative changes in the cellulose, hemicellulose, and lignin composition of the bamboo.

**Table 4:** TGA test results.

Test/sample type	Cured SS-151	Plastinated	Non-plastinated
T_5%_ (ºC)	450	90	70
T_dmax1_ (ºC)	-	270	270
T_dmax2_ (ºC)	-	330	320
T_dmax3_ (ºC)	510	440	-
Residual (%)	1	20	30

#### Flammability results

Finally, Table 5 summarizes the flammability testing results. The plastinated bamboo had a much quicker t_ignition_, and a higher TSP (Fig. 8b), SEA, and HRR (Fig. 8c). The EHC, mass loss rate (Fig. 8d), and Y_CO2_ were similar between the specimens. The values that were lower in the plastinated samples were the Y_CO_ and Peak HRR. In Fig. 8(i)-(viii), photos show the samples after ignition, gradually burning, and after testing.

**Table 5:** Summary of plastinated and non-plastinated bamboo Cone Calorimeter results over t_ignition_ + 300 s.

Test/sample type	Plastinated	Non-plastinated
t_ignition_ (s)	21 ± 3*	39 ± 4*
Peak HRR (kW/m^2^)	550 ± 80	590 ± 30
HRR_180s_ (kW/m^2^)	210 ± 30	180 ± 10
HRR (kW/m^2^)	140 ± 20	130 ± 10
EHC (MJ/kg)	15.5 ± 0.9	14.8 ± 0.3
MLR (g/s)	0.08 ± 0.02	0.075 ± 0.007
SEA (m^2^/kg)	68 ± 7*	39 ± 4*
Y_CO_ (kg/kg)	0.04 ± 0.02*	0.065 ± 0.008*
Y_CO2_ (kg/kg)	1.30 ± 0.05	1.30 ± 0.06

*Significance level, *P*-value < 0.05 (One-way ANOVA).

The time needed to establish sustainable flaming on the surface of a sample, t_ignition_, is an indication of sample flammability, as longer ignition times can sometimes prevent a fire from occurring^25,26^. Ignition occurs when enough volatiles are created that can be ignited by a spark^27^. Although the bubbles were visually not observable at the end of the plastination process, it is possible that there was some residual acetone, a highly flammable substance, left in the samples. Liquid PDMS also has a lower t_ignition_ than cured silicone^28^, thus, if there was any uncured liquid PDMS left in the samples, this would have lowered the t_ignition_. Achieving 100% cure in silicones is unlikely, at least with the current version of the plastination method.

The heat release rate can indicate the size (cascading effects) of a fire and is a very important predictor of fire hazard^26,29^. The sharp heat release rate curve indicated that the samples were relatively thin^27^. The smaller peaks present after the first peak indicate charring and heat accumulation at the back of the samples^25,27^. The HRR for most silicones is between 60 and 150 kW/m^2^, and only one of the plastinated samples had an HRR within that range. Generally, when organic volatiles are released into a flame, a higher HRR can be seen^30^. Again, this indicated that there may have been residual acetone in the plastinated samples.

SEA and TSP were used to assess smoke production. Particularly, SEA is a measure of instantaneous smoke produced per instantaneous mass loss and is presented as an average value^31^. Increased smoke, CO, and CO_2_ production during a fire can result in an increased number of casualties since they are toxic^25^. Specifically, over half of the fatalities from fires come from CO inhalation^32^. Silicone is known to produce low yields of CO^33^. Since there was more total material in the plastinated samples, i.e. silicone and bamboo, more smoke formed. Although plastinated bamboo generally had a lower flammability performance than non-plastinated bamboo, it did have a much lower CO yield.

## Conclusion

The original standard S10 plastination method for bamboo was improved in the present work, in terms of reducing both processing time and simplicity towards industrial adaptation. The improved plastination method no longer requires the use of a deep freezer during acetone dehydration, constant monitoring or high vacuum during forced polymer impregnation, or a three-part polymer that cures slowly with a spray-on agent. Instead, the method is completed at room temperature, at a lower vacuum pressure without increments, and with a one-part polymer that cures quickly with heat. It is 40% faster compared to the original method, and based on the observed Micro-CT images it yielded a 400% increase in silicone impregnation.

The flexural strength of the bamboo plastinated using the improved method was 60% stronger than non-plastinated bamboo, which was comparable to the 70% increase in tensile strength reported in^7^. Further, the specific flexural strength of the plastinated bamboo using the method was 90% greater than the virgin bamboo. Despite this mechanical performance gain, it was found that plastination using the improved method did not significantly affect the biodegradability of bamboo, likely because the process did not significantly alter the virgin material chemical composition. This was further backed-up by the FTIR and TGA results. Although the plastinated material was ignited with flame faster (due to the presence of the impregnated resin), it had a lower CO yield, and CO is the largest contributor to fire-related deaths.

The presented simplified plastination method may be assessed and further customized for other natural fibres and applications, while maintaining a high strength and biodegradability for end of-life design. Results from similar characterization tests on different natural fibers can also be eventually integrated into a multi-criteria expert system^34^ as a decision aid for designers.

## Supplementary Information

Supplementary Information.

## References

[1] Osmond R, Golovin K, Milani AS, Thomas S, Balakrishnan P (2021). Review of Chemical Treatments of Natural Fibres: A Novel Plastination Approach. Green Composites.

[2] Dwivedi AK, Kumar A, Baredar P, Prakash O (2019). Bamboo as a complementary crop to address climate change and livelihoods-Insights from India. For. Policy Econ..

[3] Clark LG, Londono X, Ruiz-Sanchez E, Clark LG (2015). Bamboo Taxonomy and Habitat. Bamboo: The Plant and its Uses.

[4] Liese W, Tang TKH, Liese W, Köhl M (2015). Properties of the Bamboo Culm. Bamboo: The plant and its uses.

[5] Dhir DK, Osmond R, Golovin K, Milani AS (2022). A high-performance hybrid green composite using plastinated bamboo fillers, with reduced environmental degradation effect. Compos. Struct..

[6] von Hagens G (1986). Plastination Folder.

[7] Dhir DK, Rashidi A, Bogyo G, Ryde R, Pakpour S, Milani AS (2020). Environmental durability enhancement of natural fibres using plastination: A feasibility investigation on bamboo. Molecules.

[8] Thomas KT, McAllister RA (1957). Densities of liquid-acetone-water solutions up to their normal boiling points. AIChE J..

[9] Ambrose D, Sprake CHS, Townsend R (1974). Thermodynamic properties of organic oxygen compounds XXXIII. The vapour pressure of acetone. J Chem Thermodyn.

[10] “Standard Test Methods for Flexural Properties of Unreinforced and Reinforced Plastics and Electrical Insulating Materials,” ASTM D790, 2017.

[11] Lipps, W., Te, B., & E, B.-H. (Eds.) 5210 Biochemical Oxygen Demand (BOD). In *Standard Methods for the Examination of Water and Wastewater*. APHA Press, 2001.

[12] Lipps, W., Te, B., & E, B.-H. (Eds.) 5220 Chemical Oxygen Demand (COD). In *Standard Methods for the Examination of Water and Wastewater*. APHA Press, 2001.

[13] *Total, Fixed, and Volatile Solids in Water, Solids, and Biosolids*, EPA-821-R-01-015 Method 1684 (2001).

[14] *Reaction-to-fire tests — Heat release, smoke production and mass loss rate-Part 1: Heat release rate (cone calorimeter method) and smoke production rate (dynamic measurement)*, ISO 5660-1 (2015).

[15] Chen H, Wu J, Shi J, Zhang W, Wang G (2021). Strong and highly flexible slivers prepared from natural bamboo culm using NaOH pretreatment. Ind. Crops Prod..

[16] Yang TC, Yang YH, Yeh CH (2020). Thermal decomposition behavior of thin Makino bamboo (*Phyllostachys makinoi*) slivers under nitrogen atmosphere. Mater. Today Commun..

[17] Rao J, Jiang J, Prosper N, Yang X, Lui T, Cai W, Wang H, Sun F (2019). Combination of polyethylene glycol impregnation and paraffin heat treatment to protect round bamboo from cracking. R. Soc. Open Sci..

[18] Wu Y, Wang Y, Yang F, Wang J, Wang X (2020). Study on the properties of transparent bamboo prepared by epoxy resin impregnation. Polymers (Basel).

[19] Pemberger N, Bittner LKH, Huck CW (2015). Using near-infrared spectroscopy to monitor the curing reaction of silicone adhesives. Spectroscopy.

[20] Xu G, Wang L, Liu J, Wu J (2013). FTIR and XPS analysis of the changes in bamboo chemical structure decayed by white-rot and brown-rot fungi. Appl. Surf. Sci..

[21] Li J, Zheng H, Sun Q, Han S, Fan B, Yao Q, Yan C, Jin C (2015). Fabrication of superhydrophobic bamboo timber based on an anatase TiO_2_ film for acid rain protection and flame retardancy. RSC Adv..

[22] Yang H, Yan R, Chen H, Lee DH, Zheng C (2007). Characteristics of hemicellulose, cellulose and lignin pyrolysis. Fuel.

[23] Makaringe NP, van der Walt IJ, Puts GJ, Crouse PL (2017). TGA-FTIR characterisation of bamboo wood, napier grass, pine wood and peach pips for gasification applications. Braz. J. Therm. Anal..

[24] Zakikhani P, Zahari R, Sultan MTH, Majid DL (2016). Thermal degradation of four bamboo species. BioResources.

[25] Xu Q, Chen L, Harries KA, Li X (2017). Combustion performance of engineered bamboo from cone calorimeter tests. Eur. J. Wood Wood Prod..

[26] Babrauskas V, Peacock DR (1992). Heat release rate: The single most important parameter in fire hazard. Fire Saf. J..

[27] Schartel B, Hull TR (2007). Development of fire-retarded materials—Interpretation of cone calorimeter data. Fire Mater..

[28] Hshieh FY, Buch RR (1997). Controlled-atmosphere cone calorimeter studies of silicones. Fire Mater..

[29] Doering H, Garche J, Liebau V, Garche KBJ (2019). Ignition and Extinction of Battery Fires. Electrochemical Power Sources: Fundamentals, Systems, and Applications.

[30] Mouritz AP, Karbhari VM (2007). Durability of Composites Exposed to Elevated Temperature and Fire. Durability of Composites for Civil Structural Applications.

[31] Adeosun, D. O., *Analysis of Fire Performance, Smoke Development and Combustion Gases from Flame Retarded Rigid Polyurethane Foams*. (2014).

[32] Gottuk DT, Roby RJ, Peatross MJ, Beyler CL (1992). Carbon monoxide production in compartment fires. J. Fire. Prot. Eng..

[33] Hamdani S, Longuet C, Perrin D, Lopez-cuesta JM, Ganachaud F (2009). Flame retardancy of silicone-based materials. Polym. Degrad. Stab..

[34] Milani AS, Shanian A, El-Lahham C (2007). A decision-based approach for measuring human behavioral resistance to organizational change in strategic planning. Math. Comput. Model..

